# Editorial for the Special Issue on Advances in Microfluidic Chips for Chemical and Biomedical Applications

**DOI:** 10.3390/mi17060733

**Published:** 2026-06-18

**Authors:** Rongxin Fu, Guoqing Hu

**Affiliations:** 1 School of Medical Science and Engineering, Beijing Institute of Technology, Beijing 100081, China; 2 Department of Engineering Mechanics, Zhejiang University, Hangzhou 310027, China

Microfluidic chips have become a central technology in modern chemical analysis, biomedical engineering, materials synthesis, and point-of-care diagnostics. By precisely manipulating fluids, particles, cells, and biochemical reactions in miniaturized spaces, microfluidic systems enable low sample consumption, rapid mass and heat transfer, high-throughput operation, and the integration of multiple analytical steps on a single platform [1,2,3]. These advantages are particularly important for applications that require automation, portability, high sensitivity, and reproducibility, including lab-on-a-chip analysis, organ-on-a-chip modeling, droplet manipulation, micro-/nanoparticle sorting, drug delivery, advanced material synthesis, and nucleic acid testing [1,2,3,4,5,6].

This Special Issue of *Micromachines*, “Advances in Microfluidic Chips for Chemical and Biomedical Applications”, provides a focused platform for recent advances in the design, fabrication, integration, and application of microfluidic technologies. The contributions include seven original research articles and four review articles, covering electrochemical lab-on-a-chip devices, digital microfluidic diagnostics, pump-free organ-on-a-chip systems, centrifugal and dielectrophoretic particle manipulation, acoustofluidic drug loading, automated platforms for *C. elegans* research, microfluidic synthesis of advanced materials, phase-change microcapsules, and lab-on-a-chip nucleic acid analysis. Collectively, these works highlight the continued evolution of microfluidics from single-function devices toward integrated, intelligent, and application-oriented platforms.

Kołczyk-Siedlecka et al. (Contribution 1) investigated the electrochemical performance of thin-film Ti-Pt microelectrodes fabricated on both planar and non-planar glass substrates for lab-on-a-chip applications. Using photolithography and physical vapor deposition, they produced microelectrodes and assessed their reproducibility through cyclic voltammetry and impedance spectroscopy. Their results demonstrated stable and repeatable electrode behavior, with only minor variations caused by substrate curvature. This work provides useful evidence for the integration of thin-film microelectrodes into complex lab-on-a-chip architectures, especially those involving non-planar geometries.

Abdalkader and Fujita (Contribution 2) developed a pump-free, 3D-printed multi-organ-on-a-chip platform for modeling the intestine–liver–muscle axis. The system uses gravity-driven oscillatory flow generated by simple rocking, avoiding the need for external pumps or complex tubing. By integrating Caco-2 intestinal epithelial cells, HepG2 hepatocyte spheroids, and primary human skeletal myoblasts, the platform supported dynamic inter-organ communication. The biological assessments showed enhanced skeletal muscle characteristics, improved hepatic function, and preserved intestinal barrier integrity. This study demonstrates the potential of accessible 3D-printed microphysiological systems for drug discovery, metabolic studies, and disease modeling.

Dongshuo Li et al. (Contribution 3) reported an automated digital microfluidic platform for antithrombin activity detection using whole capillary blood. Their device integrates droplet manipulation with optical absorbance detection and enables automated, real-time testing with minimal sample volume. By eliminating plasma separation and reducing manual operation, the system shortens the testing workflow and improves adaptability for point-of-care applications. Comparison with conventional antithrombin activity detection methods showed good consistency, supporting the clinical potential of digital microfluidics for rapid coagulation-related diagnostics.

Zekun Li et al. (Contribution 4) presented an open-type crossflow microfluidic chip for deformable droplet separation driven by a centrifugal field. The device features a wedge-shaped inlet, an elliptical chamber, and spiral microchannels, enabling size-selective droplet sorting without external pumps. By combining centrifugal force, shear stress, Dean vortices, and crossflow filtration, the chip achieved efficient separation of water-in-oil droplets. The authors also developed a theoretical model based on force balance to explain the critical separation size. This work provides a passive, structure-guided strategy for droplet sorting with potential applications in biomedical diagnostics and drug screening.

Bingxuan Li et al. (Contribution 5) introduced a MEMS-driven gigahertz acoustic streaming platform for high-efficiency drug loading in lipid vesicles. The system uses a 1.55 GHz acoustic field to generate hydrodynamic shear forces, enabling transient and nondestructive membrane permeabilization. By comparing two drug loading strategies, the authors showed that loading doxorubicin into giant unilamellar vesicles before extrusion into small unilamellar vesicles achieved superior encapsulation efficiency while preserving vesicle morphology. This study advances acoustofluidic methods for nanocarrier engineering and provides a potential route toward controllable drug delivery platforms.

Luo et al. (Contribution 6) proposed a multistage cyclic dielectrophoresis strategy for high-resolution sorting of submicron particles. The device uses a face-to-face electrode configuration to improve the uniformity of dielectrophoretic forces and reduce vertical force imbalance. By introducing repeated sorting cycles, the system reduces perturbation errors caused by Brownian motion and flow instability. The experimental results showed a significant improvement in particle monodispersity, with the coefficient of variation reduced from 12.3% to 5.4% after three sorting cycles. This contribution provides an effective microfluidic approach for the precise preparation of submicron microspheres.

Nam et al. (Contribution 7) developed a dielectrophoresis-enhanced microfluidic device with a membrane filter for efficient microparticle concentration and optical detection. The device combines the broad capture capability of a membrane filter with the spatial precision of DEP manipulation. Using transparent ITO top electrodes and Au bottom electrodes patterned on a selected membrane region, the system concentrated fluorescent polystyrene beads and *Escherichia coli* bacteria into an optimized optical detection zone. The enhanced fluorescence response indicates the potential of this platform for sensitive microparticle and bacteria detection.

Mahbub et al. (Contribution 8) reviewed automated platforms in Caenorhabditis elegans research, focusing on the integration of microfluidics, robotics, imaging, and artificial intelligence. The review summarizes foundational developments before 2020 and highlights recent advances in worm immobilization, sorting, microinjection, imaging, assay automation, and high-throughput screening. By analyzing the trade-offs among throughput, resolution, cost, and automation level, the authors provide a useful perspective on next-generation automated platforms for biological discovery using *C. elegans*.

Qi and Hu (Contribution 9) reviewed the use of microfluidics for the effective and precise synthesis of advanced materials. Their review emphasizes how microfluidic platforms provide controllable microscale reaction environments for producing polymeric, hydrogel, lipid-based, and inorganic particles with tunable size, morphology, composition, and surface chemistry. The article also discusses on-chip functionalization, process integration, AI-driven optimization, automation, and sustainable microfluidic practices. This contribution highlights the growing importance of microfluidics in advanced material design, nanomedicine, diagnostics, and personalized manufacturing.

Yuhan Li et al. (Contribution 10) reviewed microfluidics-engineered microcapsules for thermal energy storage and regulation. The review discusses how droplet microfluidics enables precise control over phase-change microcapsules, including tunable size, core–shell structure, multi-core encapsulation, and high-throughput production. The authors further summarize applications in solar energy storage, building thermal regulation, electronics cooling, and smart textiles. This contribution extends the scope of microfluidic technologies from biomedical systems to energy-related materials and thermal management.

Lee and Kim (Contribution 11) reviewed lab-on-a-chip devices for nucleic acid analysis in food safety. The review focuses on microfluidic systems for nucleic acid extraction, purification, amplification, and detection, especially those based on PCR and isothermal amplification. The authors also discuss integrated LOC devices that combine multiple analytical steps on a single chip to reduce sample preparation time and improve testing accuracy. This review demonstrates the value of portable, rapid, and sensitive microfluidic platforms for pathogen and contaminant detection in food safety applications.

Collectively, these contributions illustrate the broad and rapidly expanding impact of microfluidic chips across chemical and biomedical applications. The works in this Special Issue demonstrate that microfluidics is no longer limited to miniaturized fluid handling; rather, it is becoming an enabling platform for integrated sensing, automated diagnostics, controlled material synthesis, organ-level physiological modeling, intelligent biological experimentation, and high-throughput sample processing. The convergence of microfluidics with MEMS, electrochemistry, acoustics, dielectrophoresis, 3D printing, optical detection, robotics, and artificial intelligence will continue to drive the development of more robust, scalable, and user-friendly systems.

We sincerely thank all authors for their valuable contributions to this Special Issue. We also extend our gratitude to the reviewers for their careful evaluations and constructive comments, which helped improve the quality of the published articles. Finally, we acknowledge the editorial team of *Micromachines* for their professional support and efficient management throughout the preparation and publication of this Special Issue.
